# Radiographic evolution of a dentigerous cyst associated with a deeply impacted mandibular first molar in a pediatric patient: a CBCT-based longitudinal case report with minimal-visit orthodontic management

**DOI:** 10.1016/j.radcr.2026.05.081

**Published:** 2026-07-17

**Authors:** Huyen Nguyen Thanh, Manh Nguyen Truong, Anh Vu Dinh Viet, Hoang Viet Do, Trang Thi Thu Vu

**Affiliations:** a Department of Orthodontics, Hanoi Medical University, Hanoi, Vietnam; b Department of Oral and Maxillofacial Surgery, Hanoi Medical University, Hanoi, Vietnam; c Department of Orthodontics, National Hospital of Odonto-Stomatology, Hanoi, Vietnam

**Keywords:** Dentigerous cyst, CBCT, Impacted molar, Marsupialization, Skeletal anchorage

## Abstract

A deeply impacted mandibular first molar associated with a dentigerous cyst is an uncommon clinical presentation, particularly in pediatric patients. This case report describes a 9-year-old boy who presented with delayed eruption of the mandibular right first molar. Panoramic radiography revealed a well-defined unilocular radiolucent lesion associated with a deeply impacted tooth, while cone-beam computed tomography (CBCT) demonstrated buccolingual cortical expansion and thinning, displacement of the adjacent second molar germ, and close proximity to the inferior alveolar canal. Based on clinical, radiographic, and histopathologic findings, the lesion was diagnosed as a dentigerous cyst and treated conservatively with marsupialization followed by orthodontic traction. Longitudinal radiographic follow-up over 16 months demonstrated progressive reduction of the radiolucent lesion, gradual bone regeneration, and continuous eruption and uprighting of the impacted mandibular first molar. This case highlights the important role of CBCT and serial radiographic monitoring in evaluating lesion extent, treatment planning, and documenting healing and tooth eruption during conservative management of dentigerous cysts in children.

## Introduction

Dentigerous cysts are most frequently associated with impacted mandibular third molars, maxillary canines, and mandibular second molars. In contrast, association with a deeply impacted mandibular first molar is rare, particularly when accompanied by severe displacement and delayed eruption requiring combined surgical-orthodontic management [[1], [2], [3], [4], [5]]. These lesions are frequently asymptomatic and are often discovered incidentally during routine radiographic examinations [3,6,7]. Radiographically, dentigerous cysts typically present as well-defined, unilocular radiolucencies attached at the cemento-enamel junction of the involved tooth, although variations in size and extent may occur [6,[8], [9], [10]].

Panoramic radiography is widely used as the initial imaging modality for the detection of such lesions due to its accessibility and broad coverage [8,11]. However, it has inherent limitations in accurately depicting the three-dimensional extent of lesions and their spatial relationship with adjacent anatomical structures [12,13]. Cone-beam computed tomography (CBCT) has emerged as a valuable adjunct imaging modality, offering high-resolution three-dimensional visualization that allows precise assessment of lesion boundaries, cortical bone integrity, and displacement of adjacent teeth and neurovascular structures [13,[14], [15], [16], [17], [18], [19], [20], [21]].

In pediatric patients, accurate imaging evaluation is particularly critical because ongoing craniofacial growth and the presence of developing tooth germs may complicate both diagnosis and treatment planning [17,[22], [23], [24]]. Dentigerous cysts in this population may lead to delayed eruption, displacement of adjacent teeth, and cortical expansion, making early detection and longitudinal monitoring essential [18,13,25].

The present report describes the radiographic characteristics and longitudinal imaging evolution of a dentigerous cyst associated with a deeply impacted mandibular first molar in a pediatric patient. Emphasis is placed on the diagnostic value of CBCT and the role of serial radiographic follow-up in evaluating treatment response and bone regeneration.

## Case presentation

### Clinical findings

A 9-year-old boy was referred with a chief complaint of delayed eruption of the lower right posterior tooth. The patient reported no associated pain or swelling, and his medical history was non-contributory.

Extraoral examination revealed a symmetrical facial profile with no evidence of swelling or deformity. Intraoral examination demonstrated mixed dentition. The mandibular right first molar was clinically absent, and the overlying mucosa appeared normal in color and texture. The mandibular right second molar exhibited incomplete root development without signs of dental caries or pathology (Fig. 1).

**Fig. 1:**
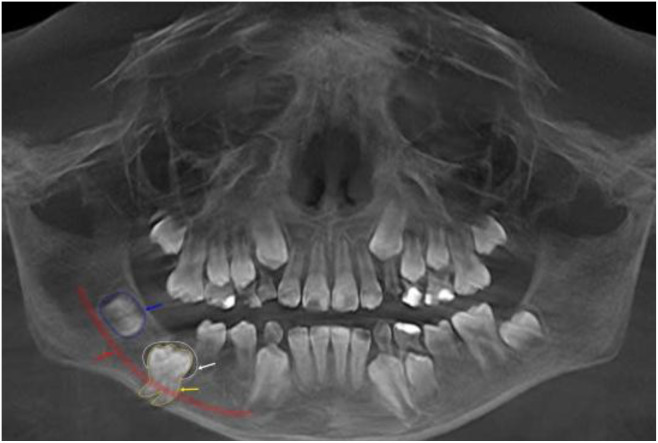
Pretreatment panoramic radiograph. Initial panoramic radiograph and CBCT images demonstrating a well-defined cystic radiolucent lesion associated with a deeply impacted mandibular right first molar. CBCT images reveal buccolingual cortical expansion with cortical thinning and medial displacement of the inferior alveolar canal while preserving canal cortication. The developing mandibular right second molar germ is displaced distally by the lesion. Arrows indicate the cystic lesion margin, impacted first molar, displaced inferior alveolar canal, and displaced second molar germ. White arrow: cystic lesion margin. Yellow arrow: deeply impacted mandibular right first molar. Red arrow: medially displaced inferior alveolar canal. Blue arrow: displaced mandibular right second molar germ.

### Detailed radiologic description

Panoramic radiography revealed a well-defined, unilocular radiolucent lesion in the right mandibular body, associated with the crown of a deeply impacted mandibular first molar [6,8,9]. The lesion demonstrated a well-defined corticated radiolucent margin enveloping the crown of the impacted tooth, consistent with the imaging characteristics of a dentigerous cyst. The lesion extended from the distal aspect of the mandibular second premolar region posteriorly toward the ramus, producing marked expansion and displacement of the associated developing teeth.

The mandibular right first molar was displaced inferiorly toward the inferior mandibular border, with significant disruption of its normal eruption trajectory. The tooth exhibited a predominantly vertical orientation with lingual inclination. Root development was nearly complete, although apical closure had not yet occurred. The adjacent developing tooth germ was displaced superiorly and posteriorly into a position simulating a third molar. Given the absence of third molars in all 4 quadrants, this ectopic tooth germ was identified as the mandibular right second molar (Fig. 2) [13,18,23].

**Fig. 2:**
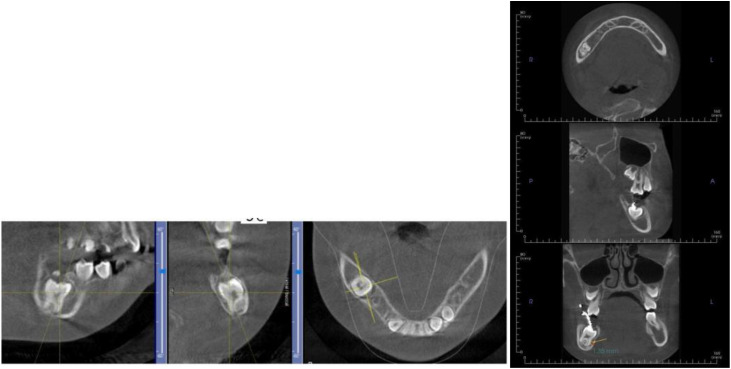
Cone-beam computed tomography (CBCT) images at initial presentation. (A) Axial view showing a well-corticated radiolucent lesion with buccolingual expansion and thinning of the cortical plates. (B) Coronal view demonstrating inferior and lingual displacement of the impacted mandibular first molar. (C) Sagittal view illustrating the relationship between the cystic lesion and the inferior alveolar canal, which is displaced but remains intact.

CBCT imaging further characterized the lesion as a cystic expansile radiolucency centered around the crown of the deeply impacted mandibular right first molar. Multiplanar reconstruction demonstrated buccolingual cortical expansion with cortical thinning, particularly along the buccal cortex, without definite cortical perforation. The lesion displaced the inferior alveolar canal medially, with the canal coursing closely along the inferior border of the cystic cavity. Despite displacement, cortication of the inferior alveolar canal remained preserved. Axial and cross-sectional CBCT views clearly demonstrated the three-dimensional spatial relationship between the lesion, impacted tooth, cortical plates, and inferior alveolar canal, which was essential for surgical and orthodontic treatment planning (Fig. 3.).

**Fig. 3:**
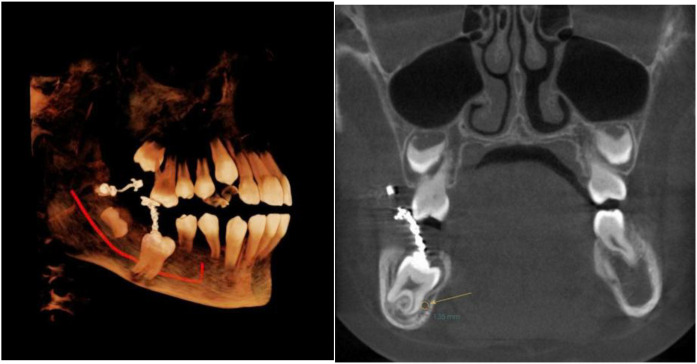
Cone-beam computed tomography (CBCT) images of relationship between the cyst and the inferior alveolar nerve canal.

The cystic lesion extended around the crown and cervical region of the impacted first molar and contributed to displacement of the adjacent developing second molar germ. Serial radiographic follow-up demonstrated progressive decompression of the lesion, gradual bone regeneration, and favorable eruption movement following marsupialization and orthodontic traction.

CBCT was essential for evaluating the three-dimensional relationship among the impacted mandibular first molar, cystic cavity, cortical plates, and inferior alveolar canal. Imaging findings demonstrated medial displacement of the inferior alveolar canal and significant buccolingual osseous expansion, features that could not be fully appreciated on panoramic radiography alone [11,14,17,19].

### Management and follow-up

The lesion was managed conservatively by marsupialization, with the aim of reducing intracystic pressure, promoting gradual decompression, and facilitating bone regeneration. The primary treatment objective was to address the patient’s chief complaint of delayed eruption of the mandibular right molars. Accordingly, the treatment plan focused on guided eruption of the impacted teeth into their appropriate positions within the dental arch while simultaneously reducing the associated osseous defect. Additional objectives included improvement of occlusion, establishment of a Class I molar and canine relationship, and correction of the dental midline deviation.

Treatment was performed in collaboration with the Department of Oral and Maxillofacial Surgery. Following elevation of a mucoperiosteal flap, the superior portion of the cystic lesion was excised to create a marsupialization window and allow continuous decompression of the lesion. During the procedure, a biopsy specimen was obtained, and histopathological examination confirmed the diagnosis of a dentigerous cyst [12,22,[26], [27], [28]].

Orthodontic traction of the impacted molars was initiated after marsupialization using a C-plate anchorage system combined with elastic thread traction. Light continuous orthodontic force, approximately 50 g, was initially applied to minimize the risk of damage to the developing teeth and surrounding structures. The traction force was gradually adjusted in accordance with continued root development and progressive apical maturation. Continuous elastic traction facilitated gradual eruptive movement of the impacted teeth toward the occlusal plane over time.

To further illustrate the developmental displacement pattern and spatial relationship among the impacted mandibular first molar, displaced second molar germ, inferior alveolar canal, and cystic expansion, a schematic diagram was created based on the CBCT findings (Fig. 4, Fig. 5).

**Fig. 4:**
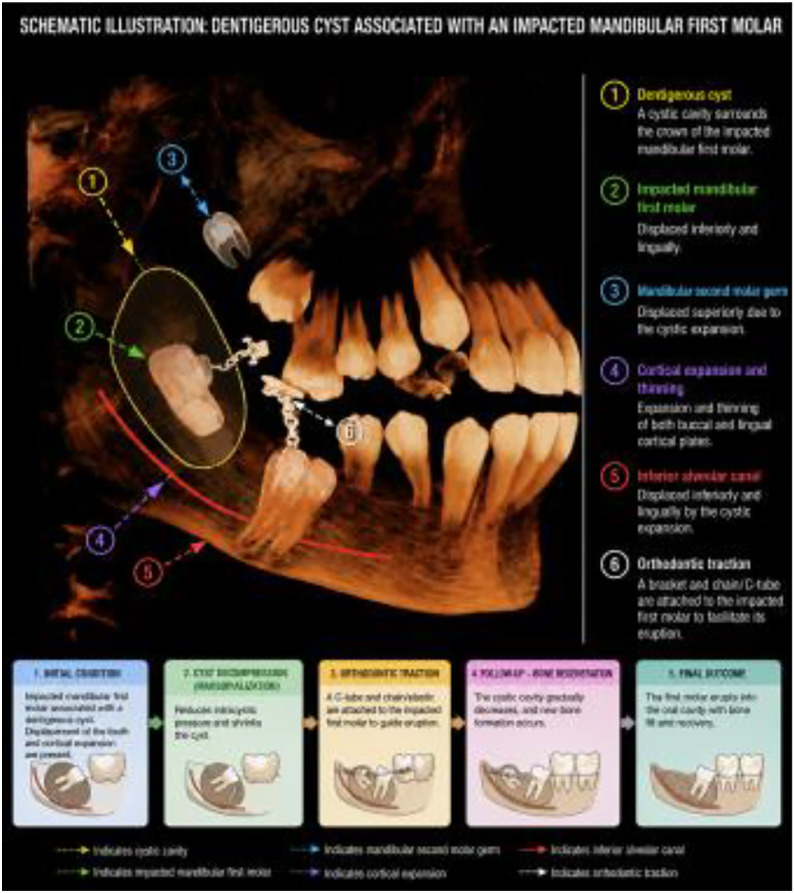
Schematic illustration of a dentigerous cyst associated with a deeply impacted mandibular right first molar in a pediatric patient**.** The diagram demonstrates inferior and lingual displacement of the impacted first molar, superior displacement of the mandibular second molar germ, cortical expansion and thinning, displacement of the inferior alveolar canal, and the longitudinal treatment sequence including marsupialization, orthodontic traction, and bone regeneration.

**Fig. 5:**
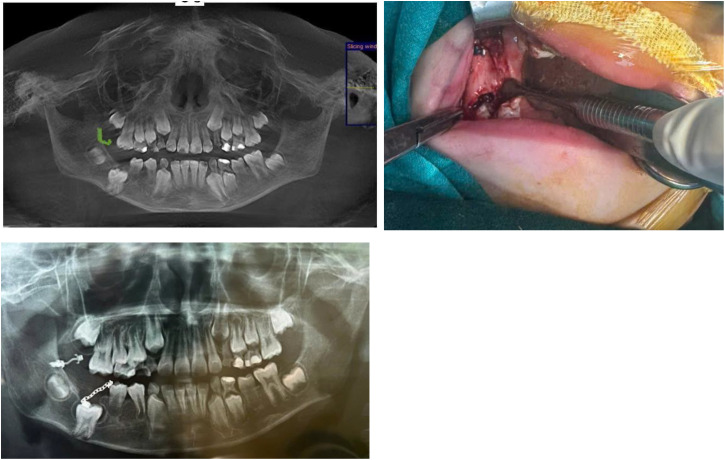
Early follow-up panoramic radiograph after marsupialization. Panoramic radiograph demonstrating partial reduction in lesion size and early signs of bone regeneration. The impacted molar shows initial movement toward the occlusal plane.

Because the patient resided far from the treatment center, regular monthly follow-up appointments were not feasible. Consequently, clinical and radiographic follow-up visits were scheduled at intervals of approximately 3–4 months. Despite the relatively infrequent follow-up, serial radiographs demonstrated progressive reduction of the cystic radiolucency, gradual bone regeneration, and continued eruption of the impacted molars [2,29,30].

Serial radiographs were obtained at regular intervals during the follow-up period. Progressive reduction in lesion size and gradual bone regeneration were observed. The impacted molar demonstrated gradual eruption toward the occlusal plane, facilitated by both spontaneous and assisted movement [15,16,25,[31], [32], [33]] (Fig. 5, Fig. 6).

**Fig. 6:**
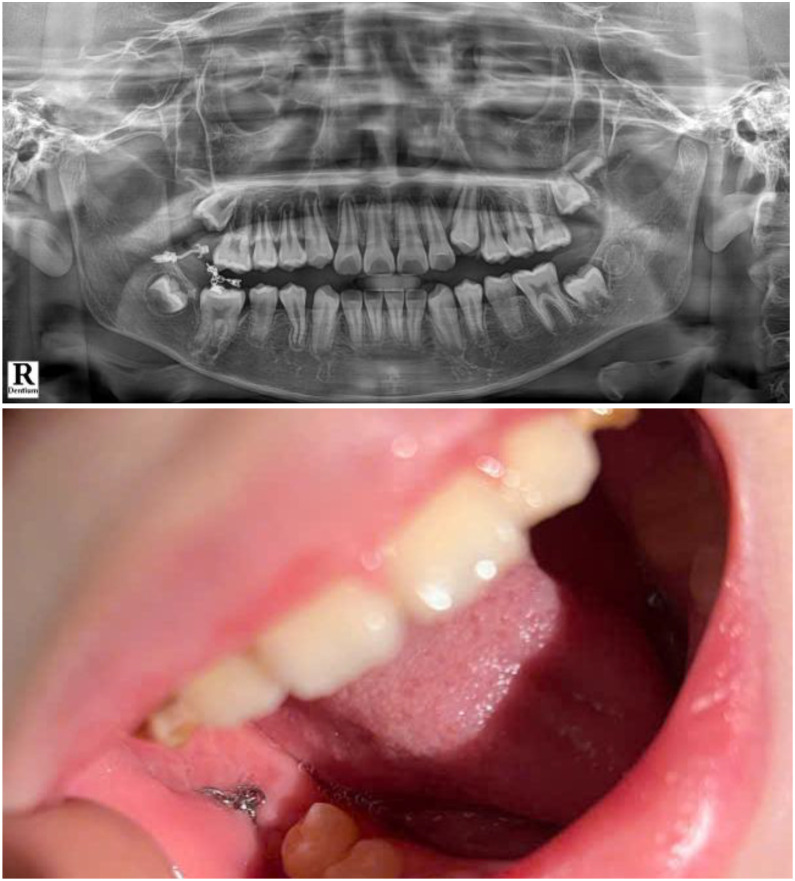
Final panoramic radiograph at 16-month follow-up. October 23, 2025. Panoramic radiograph demonstrating near-complete resolution of the radiolucent lesion with satisfactory bone fill. The mandibular right first molar has erupted into a functional vertical position.

At 16 months, the first molar had erupted into a functional vertical position. Radiographic evaluation demonstrated near-complete resolution of the radiolucent lesion with satisfactory bone fill.

**Table:** 

Follow-up interval	Radiographic findings	Bone regeneration	Position of impacted molar
Initial presentation	Large pericoronal radiolucent lesion associated with the mandibular right molar, causing marked tooth displacement	Significant bone loss involving the posterior mandibular body and angle region	Impacted molar positioned close to the inferior mandibular border
Intermediate follow-up	Gradual reduction in lesion radiolucency and decrease in lesion size	Progressive increase in bone density within the affected region	Impacted molar showed superior eruptive movement toward the occlusal plane
Most recent follow-up	Marked reduction of the radiolucent area	More evident trabecular bone formation within the previous defect	Continued eruption and improvement in tooth angulation

## Discussion

Dentigerous cysts are typically diagnosed based on their characteristic radiographic presentation as well-defined unilocular radiolucencies associated with the crown of an unerupted tooth [2,6,8,4,9].However, several odontogenic lesions may present with overlapping radiographic features in pediatric patients, making differential diagnosis essential [34]. Deepa et al. emphasized the importance of correlating clinical, radiographic, and histopathologic findings to distinguish dentigerous cysts from other odontogenic entities with similar imaging appearances. While this appearance is often considered pathognomonic, variations in lesion size, tooth displacement, and cortical involvement may complicate diagnosis, particularly in pediatric patients with developing dentition.

Panoramic radiography remains the primary screening tool due to its accessibility and ability to provide a global overview of the dentition [8,11]. However, its inherent limitations in assessing three-dimensional relationships and subtle cortical changes have been well documented [12,13]. In the present case, panoramic imaging was sufficient to identify the lesion but inadequate for accurately characterizing its full extent and its relationship to adjacent anatomical structures.

CBCT provided critical additional information by enabling precise evaluation of cortical expansion, buccolingual involvement, and displacement of the inferior alveolar canal [[13], [14], [15], [16], [17], [18], [19], [20], [21]]. This three-dimensional assessment was particularly important in determining the direction of displacement of the impacted molar and the spatial relationship of the adjacent tooth germ. Such information is essential not only for diagnosis but also for guiding treatment planning and minimizing the risk of iatrogenic injury.

A notable diagnostic challenge in this case was the superior displacement of the adjacent tooth germ into a position resembling that of a third molar. Differentiating between ectopic eruption, developmental anomalies, and true displacement due to cystic expansion requires careful integration of imaging findings with developmental staging [13,18,23,24]. The absence of third molars in all quadrants, combined with the stage of root development, supported the identification of this structure as the mandibular second molar. This highlights the importance of developmental context in radiologic interpretation, particularly in pediatric populations.

From a therapeutic perspective, marsupialization remains a well-established conservative approach for the management of dentigerous cysts, especially in young patients [4,22,[26], [27], [28],^35^]. Recent evidence from a systematic review by Cobo-Vázquez et al. further supports the effectiveness of conservative treatment approaches in pediatric patients, particularly in promoting spontaneous eruption and preservation of developing teeth [36]. By reducing intracystic pressure, this technique promotes bone regeneration and may facilitate eruption of the associated tooth. The present case demonstrated progressive reduction in lesion size and gradual bone formation on serial imaging, consistent with previously reported outcomes.

Beyond the conventional management approach, an important distinguishing feature of this case is the integration of skeletal anchorage to facilitate orthodontic traction with minimal reliance on patient compliance. Skeletal anchorage systems, including miniplates and C-tube devices, have been shown to provide stable force application and effective tooth movement, particularly in complex impaction cases [[37], [38], [39], [40], [41], [42]]. In contrast to conventional orthodontic mechanics, these systems reduce unwanted reciprocal forces on adjacent teeth and allow for more controlled tooth movement.

Importantly, the use of a C-tube anchorage system in this case enabled the application of light, continuous force while significantly reducing the need for frequent clinical adjustments. This aspect is particularly relevant in the context of patients from remote or underserved areas, where access to specialized orthodontic care may be limited. The combination of skeletal anchorage and simplified biomechanics represents a pragmatic and efficient treatment strategy that aligns with real-world clinical constraints.

Another key strength of this report lies in the longitudinal radiographic follow-up, which provided objective documentation of treatment response over time. Serial imaging demonstrated not only cyst resolution but also progressive bone regeneration and functional tooth eruption. This reinforces the role of imaging as an essential tool not only for diagnosis but also for dynamic monitoring of treatment outcomes.

Despite these strengths, certain limitations should be acknowledged. As a single case report, the findings may not be generalizable to all clinical scenarios. Additionally, while CBCT offers significant diagnostic advantages, its use should be carefully justified, particularly in pediatric patients, due to radiation considerations. Future studies with larger cohorts are needed to further evaluate the combined role of CBCT and skeletal anchorage systems in the management of odontogenic cysts.

Overall, this case underscores the evolving role of advanced imaging and minimally dependent orthodontic mechanics in the management of dentigerous cysts. The integration of CBCT-based evaluation with low-frequency follow-up strategies may represent a valuable approach for optimizing outcomes while reducing treatment burden in selected patient populations.

## Conclusion

Imaging plays a central role in the diagnosis and management of dentigerous cysts. While panoramic radiography is effective for initial detection, CBCT provides essential three-dimensional information that enhances diagnostic accuracy and supports appropriate treatment planning [17,19,20].

Longitudinal radiographic follow-up is critical for assessing lesion regression and bone regeneration. Conservative management, when guided by comprehensive imaging evaluation, can result in favorable clinical and radiographic outcomes, particularly in pediatric patients.

## Author contributions

**Trang Thi Thu Vu:** Conceptualization, Methodology, Investigation, Writing—Original Draft, Visualization. **Nguyen Truong Manh:** Surgical management, Supervision. **Nguyen Thanh Huyen, Do Hoang Viet, and Vu Dinh Viet Anh:** Writing—Review and Editing.

## Data availability statement

The data supporting the findings of this study are available from the corresponding author upon reasonable request.

## Ethical approval

This study was conducted in accordance with the Declaration of Helsinki. Ethical approval was obtained from the institutional review board of Hanoi Medical University.

## Patient consent

Written informed consent was obtained from the patient’s legal guardian for publication of this case report and accompanying images. A copy of the written consent is available for review by the Editor-in-Chief of this journal on request.
